# The CYP2J2 G-50T polymorphism and myocardial infarction in patients with cardiovascular risk profile

**DOI:** 10.1186/1471-2261-8-41

**Published:** 2008-12-23

**Authors:** Jan Börgel, Daniel Bulut, Christoph Hanefeld, Horst Neubauer, Andreas Mügge, Jörg T Epplen, Tim Holland-Letz, Martin Spiecker

**Affiliations:** 1 Department of Cardiology, St. Josef Hospital/Bergmannsheil, Ruhr-University, Bochum, Germany; 2 Department of Human Genetics, Ruhr-University, Bochum, Germany; 3 Department of Medical Informatics, Biometry and Epidemiology, Ruhr-University, Bochum, Germany; 4 Department of Cardiology, Marienhospital, Marl, Germany

## Abstract

**Background:**

Cytochrome P450 (CYP) enzyme 2J2, an epoxygenase predominantly expressed in the heart, metabolises arachidonic acid to biologically active eicosanoids. One of the CYP2J2 products, 11, 12-epoxyeicosatrienoic acid, has several vasoprotective effects. The CYP2J2-G-50T-promotor polymorphism decreases gene expression and is associated with coronary artery disease. This association supports the vascular protective role of CYP-derived eicosanoids in cardiovascular disease. In the present study, we investigated the influence of this polymorphism on survived myocardial infarction in two study groups of patients with on average high cardiovascular risk profile.

**Methods:**

The CYP2J2 polymorphism was genotyped in two groups of patients that were collected with the same method of clinical data collection. Data from 512 patients with sleep apnoea (group: OSA) and on average high cardiovascular risk profile and from another 488 patients who were admitted for coronary angiography (CAR-group) were evaluated for a potential correlation of the CYP2J2 polymorphism G-50T and a history of myocardial infarction. The G-50T polymorphism of the CYP2J2 gene was genotyped by allele specific restriction and light cycler analysis.

**Results:**

The T-allele of the polymorphism was found in 111 (11.1%; CAR-group: N = 65, 13.3%; OSA: N = 46, 9.0%). 146 patients had a history of myocardial infarction (CAR: N = 120, 24.6%; OSA: N = 26, 5.1%). Cardiovascular risk factors were equally distributed between the different genotypes of the CYP2J2 G-50T polymorphism. In the total group of 1000 individuals, carriers of the T-allele had significantly more myocardial infarctions compared to carriers of the wild type (T/T or G/T: 21.6%; G/G: 13.7%; p = 0.026, odds ratio 1.73, 95%-CI [1.06–2.83]). In the multivariate logistic regression analysis the odds ratio for a history of myocardial infarction in carriers of the T-allele was 1.611, 95%-CI [0.957–2.731] but this trend was not significant (p = 0.073).

**Conclusion:**

In presence of other risk factors, the CYP2J2 G-50T failed to show a significant role in the development of myocardial infarction. However, since our result is close to the border of significance, this question should be clarified in larger, prospective studies in the future.

## Background

The human Cytochrome P450 enzyme, CYP2J2, is abundantly expressed in coronary artery endothelial and smooth muscle cells as well as in cardiac myocytes [1]. Cytochrome P450 epoxygenases metabolise arachidonic acid to epoxyeicosatrienoic acids (EETs). These eicosanoids exert anti inflammatory [2-4], vasodilator, [5-7], antithrombotic, antimigratory, antioxidant, and antiapoptotic effects [3,8].

The CYP2J2 G-50T polymorphism is a relatively frequent polymorphism that was observed in the proximal promoter region of the gene, which causes a loss of transcription factor binding site Sp1. This leads to a reduced interaction of this transcription factor with the CYP2J2 promoter. In vascular endothelial cells, the G-50T polymorphism was associated with a 50% reduction in CYP2J2 promoter activity compared with that of the wild type promoter. As a consequence, individuals with the G-50T polymorphism had significantly lower plasma concentrations of 14,15-DHET (dihydroxeicosatrienoicacids, the measurable, stable metabolite of 14, 15-EET), compared with wild type individuals [9]. The relevance of these findings for human atherosclerosis was assessed in a cohort of N = 500 patients (age limit, <65 years) with and without coronary artery disease (CAD). Individuals with angiographic exclusion of CAD had a homozygous or heterozygous CYP2J2 promoter polymorphism in 10.6%, while it was found in a significantly higher proportion of 17.3% in individuals with coronary stenosis > 50. After adjustment for conventional risk factors, age and sex, the odds ratio for having CAD was still significantly higher for carriers of the T allele (odds ratio: 2.23) [9]. It is suggested that the CYP2J2 dependent anti inflammatory, anti thrombotic and vasodilatative mechanisms may also play a role in the development of myocardial infarctions. However, the studies so far published yielded conflicting results regarding the correlation of the G-50T polymorphism and myocardial infarction [10,11]

The present study investigates, whether the CYP2J2 G-50T polymorphism promotes the development of myocardial infarctions in patients with high cardiovascular risk profile.

## Methods

### Patients

The Polymorphism was genotyped in two study groups of patients that were collected at the University of Bochum in order to investigate predisposing genetic factors for the development of cardiovascular risk factors, coronary heart disease and myocardial infarction. The data collection was performed with the same patient's questionnaire and the same design of the ACCESS-database. Therefore the cut off values and criteria for the determination of the different risk factors were the same in both groups. Dyslipidemia was diagnosed by patient recall and when patients were either taking lipid lowering drugs or had dyslipidemic serum level values. For blood analysis, the following cut-off values were defined as abnormal: total cholesterol ≥ 200 mg/dl, triglycerides ≥ 180 mg/dl, HDL-C ≤ 40 mg/dl, and LDL-C ≥ 150 mg/dl. Diabetes was defined by patient recall and also if patients were taking antidiabetic medication or insulin therapy. The diagnosis hypertension was established if patients were either on antihypertensive medication or if the mean of three measured systolic BP exceeded 140 mmHg or the diastolic BP 90 mmHg, respectively. The diagnosis myocardial infarction was established by patient recall and was confirmed by viewing the patient records (i.e. ECG, echocardiography).

All patients gave informed consent and explicitly provided permission for DNA analysis and collection of relevant clinical data. The study protocols were approved by the local ethics committee (University of Bochum, Germany).

Patients in the obstructive sleep apnea study group (OSA group, N = 512) were consecutively enrolled during the years 1999 to 2001 in the sleep laboratory of the Marienhospital, Herne, Ruhr-University Bochum, Germany. The patients were admitted to the hospital because of a history of apneas, snoring or hypersomnic symptoms like day time tiredness or impairment of cognitive functions. Detailed information about the determination of cardiac risk factors and polysomnography were described extensively in previous publications [12,13] The subjects of the other group (CAR group, N = 488) were consecutive patients encountered in the cardiac catheterisation laboratory in the St. Josef Hospital of the Ruhr-University Bochum from October 2001 to August 2002. The complete clinical history, including cardiovascular risk factors, was obtained from all study subjects by use of the same data collection method as in the OSA group (see above).

### Genotyping

Patient DNA was isolated from whole-blood samples by the phenolchloroform extraction method. The promoter polymorphism G-50T was genotyped by a restriction endonuclease digestion system. The G-50T polymorphism creates a novel restriction site for the AluI endonuclease (recognition sequence AGCT). PCR (using the following primers: sense, 5-TTTTCTGAGACCGGTGCGTG-3; antisense: 5-TAGGAGAGTCCGAGGATGGA-3) yielded a 242-bp product including the polymorphism. Incubation with AluI resulted in 2 fragments (99 and 143 bp) in the PCR products, containing the T-allele, but not in those with the wild type. Results of restriction analysis were confirmed by Light-Cycler PCR using the following PCR-primers and the following specific sensor and anchor primers:

**Forward-Primer: **5' TGCGTGTCCTCCCGGGAAT 3'

**Reverse-Primer: **3 GGAGAGZCCGAGGATGGACCA 5'

Results of restriction analysis were confirmed by Light-Cycler PCR using the following specific sensor and anchor primers:

**Anchor: **CTCGCTGGTGTTCATCTTTGGTTTTGT

**Sensor: **CAACATGCTGGTCATCCTCATCTTAAT

The genotypes of the CYP2J2 G-50T polymorphism were identified by the different melting temperatures for the G (61°C) and the T allele (66°C).

### Statistical analysis

Results are presented as means +/-SD. All reported P-values are two-tailed. Statistical analyses were performed with the computer software SPSS for Windows (SPSS, Chicago, IL, USA). Demographic characteristics of the patients were compared between the different genotypes by using a chi square test and were considered significantly different if the p-values were <0.05. Because of the rare incidence of homocygote T-allele carriers, heterocygote and homocygote T-allele genotypes of the CYP2J2 G-50T polymorphism were pooled to one group.

Comparisons between the CYP2J2 G-50G group and the CYP2J2 G-50T/T-50T group were performed by students t-test for unpaired samples.

The influence of the T-allele (G-50T/T-50T) on myocardial infarction was then analysed in a multivariate logistic regression model, adjusted for diabetes, dyslipidemia, hypertension, gender, smoking and age.

## Results

The T-allele of the polymorphism was found in 111 (11.1%, CAR: N = 65, 13.3%; OSA: N = 46, 9.0%). Cardiovascular risk factors were equally distributed between the different genotypes of the CYP2J2 G-50T polymorphism (Table 1).

**Table 1:** Cardiovascular risk factors in the different genotypes of the CYP2J2 G-50T polymorphism

		**N (%)**	**Age ± SD**	**BMI ± SD**	**Hypert. N (%)**	**Diabet. N (%)**	**Dyslipid. N (%)**	**Smoking** **N (%)**
**OSA-group** **N = 512**	*All*	512	55.4 ± 10.6	30.9 ± 5.7	274 (53.2)	77 (15.0)	219 (42.8)	288 (71.6)
	*G/G*	466 (91.0)	55.0 ± 10.6	30.8 ± 5.7	246 (52.8)	68 (14.6)	197 (42.3)	259 (71.0)
	*G/T T/T*	46 (9.0)	58.3 ± 9.6	31.2 ± 5.6	28 (60.9)	9 (19.6)	22 (47.8)	29 (78.1)
	*P*		0.045	0.621	0.295	0.368	0.468	0.34
								
**CAR-group N = 488**	*All*	488	65.1 ± 11.3	27.7 ± 4.1	368 (75.4)	116 (23.8)	299 (61.3)	329 (67.4)
	*G/G*	423 (86.7)	65.3 ± 11.3	27.8 ± 4.2	321 (75.9)	100 (23.6)	261 (61.7)	282 (66.7)
	*G/T T/T*	65 (13.3)	63.5 ± 11.2	27.0 ± 4.1	47 (72.3)	16 (24.6)	38 (58.5)	47 (72.3)
	*P*		0.240	0.153	0.533	0.854	0.618	0.36
								
**All N = 1000**	*All*	1000	60.1 ± 12.0	29.2 ± 5.1	642 (64.2)	193 (19.3)	518 (51.8)	617 (69.3)
	*G/G*	889 (88.9)	59.9 ± 12.1	29.3 ± 5.2	567 (63.8)	168 (18.9)	458 (51.5)	541 (68.7)
	*G/G T/T*	111 (11.1)	61.4 ± 10.8	28.6 ± 6.1	75 (67.6)	25 (22.5)	60 (54.1)	76 (74.5)
	*P*		0.220	0.227	0.433	0.362	0.614	0.23

Abbreviations: OSA-Patients: Patients with obstructive sleep apnea, CA-Patients: patients, referred to coronary angiography, BMI: body mass index, Hypert: Hypertension, Diabet.: diabetes, Dyslipid: dyslipidemia,

In the total group 146 patients had a history of myocardial infarction (CAR: N = 120, 24.6%; OSA: N = 26, 5.1%). In the t-test for unpaired samples the group of patients carrying the T-allele had significantly more myocardial infarctions compared to carriers of the wild type (T/T or G/T: 21.6% vs. G/G: 13.7%; chi-square-test: p = 0.026; odds ratio: 1.73, 95%-CI [1.06–2.83] Table 2).

**Table 2:** History of myocardial infarction in the different genotypes of the CYP2J2 G-50T polymorphism

	**OSA-Group** **N = 512**		**CAR-Group** **N = 488**		**All** **N = 1000**	
	**G/G**	**G/T or T/T**	**G/G**	**G/T or T/T**	**G/G**	**G/T or T/T**
*N*	466	46	423	65	889	111
*MI*	21 (4.5%)	5 (10.9%)	101 (23.9%)	19 (29.2%)	122 (13.7%)	24 (21.6%)
P	0.061		0.351		0.026	

Abbreviation: MI = Myocardial infarction.

A multivariate logistic regression analysis between the risk factors and myocardial infarction could only include 890 patients because of missing or invalid information about smoking habits in 110 patients. After multivariate regression the odds ratio for myocardial infarction in carriers of the T allele slightly decreased 1.611, 95%-CI [0.957–2.731]. However, although other risk factors did not differ between the genotypes, the p value of this relationship barely exceeded the border of significance (p = 0.073) as shown in Table 3.

**Table 3:** Multivariate logistic regression between risk factors and myocardial infarction

	***Myocardial Infarction adjusted***	
	**Odds ratio (Confidence Interval)**	**p**
**CYP2J2 G/T or T/T**	1.611 (0.957 – 2.731)	0.073
**Diabetes**	1.529 (0.995 – 2.349)	0.052
**Hypertension**	1.126 (0.725 – 1.748)	0.597
**Dyslipidemia**	1.644 (1.100 – 2.455)	0.015
**Age**	1.049 (1.030 – 1.069)	<0.001
**Smoking**	1.622 (1.233 – 2.125	<0.001
**Gender**	1.311 (0.844 – 2.099)	0.219

## Discussion

We studied the influence of the CYP2J2 G-50T polymorphism on the history of myocardial infarction in two study groups. In order to achieve a sufficient number of patients, a pooled analysis from 2 different study groups was performed. Both study groups represent patients with high cardiovascular risk profiles. The same criteria for cardiovascular risk factor classification were used in these groups and genotyping was performed with the same methods. Thus, of 1000 patients, a relative high number of 146 patients had a history of myocardial infarction. As expected, the OSA group showed a lower rate of myocardial infarction than the CAR group which was collected in the catheterization laboratory of a cardiology unit. However, the cardiovascular risk factor profile of OSA patients is similar to those patients presenting in hospital with suspected coronary heart disease. In addition, several studies have shown that OSA itself aggravates conventional risk factors for myocardial infarction, especially arterial hypertension [13,14] dyslipidemia [12], and insulin resistance [15]. Further, OSA independently increases the risk of sudden fatal and non fatal cardiovascular events [16]. This may be the reason for the relative high percentage of myocardial infarctions (5.1%) in the comparatively young OSA group with an age of on average 55 years.

Although multivariate regression analysis could not show a significant relation between myocardial infarction and the G-50T polymorphism, our results do not definitely exclude a role of this polymorphism in the pathogenesis of myocardial infarction. Importantly, approximately 30% or more of those individuals with myocardial infarction die before hospital admission. Therefore a definite answer regarding the potential role of the CYP2J2 promoter polymorphism in this regard can only be achieved by prospective analysis of a cohort with an adequate follow-up period.

The CYP2J2 G-50T polymorphism via lower EET serum levels may negatively influence many pathways which are suspected to cause myocardial infarction. Since the polymorphism has been detected recently, there are currently few population-based studies about the association with myocardial infarction. However, in a recent study, Liu and co workers found significantly more carriers of the T-allele in a group of 200 young patients with acute myocardial infarction, compared with a group of age matched controls [11]. In addition they could demonstrate *in vitro *that in cells with the T-allele but not in the wild type cells, nicotine significantly reduced promoter activity. EET metabolites were significantly lower among CYP2J2 T-allele carriers, compared to carriers of the wild type.

Smoking and this variant had a significant interactive and synergistic effect on the development of myocardial infarction.

In contrast to the above mentioned results we could not show an independent synergistic interaction between the T-allele, smoking and myocardial infarction. One important reason for this may be the younger age in the study group of Liu et al. It is well known that genetic predisposition in cardiovascular disease is predominantly relevant in younger age [17].

Another study could not confirm a correlation between the G-50T promoter polymorphism and myocardial infarction in a large cohort of patients [10]. The reason for these conflicting results remain at least in part unclear, as in other genotype association studies. One possible explanation are differences in the methods used. Our previous experience has shown that genotyping with the restriction endonuclease method (using the enzyme AluI) is unreliable in some cases. Light cycler analysis in the case of the G-50T polymorphism is more reliable compared to direct sequencing. Given the relative small number of individuals with the polymorphism, the method of genotyping used might contribute to different results.

As mentioned above, genetic predisposition to myocardial infarction is predominantly relevant in younger age [17]. While Liu et al. [11] found significant results in a younger study group (age < 45), the insignificant results of the LURIC Study [10] were collected in group with a mean age of 64 years. The same analysis in our collective with a mean age 58 years generates a result at the border of significance. Therefore the level of significance may indeed correlate inversely with the age of the study group. This may be another reason for the different results on the genetic impact of the CYP2J2 G-50T polymorphism on myocardial infarction.

Observing the overall group of 1000 patients, a history of myocardial infarction is the only phenotype showing a significant difference between the genotypes of the investigated polymorphism. Although the results of the multivariate analysis are not significant, this trend, in our view, may indicate a role of the CYP2J2 G-50T polymorphism with its anti-inflammatory, vasodilatative and antithrombotic relevance in the genesis of myocardial infarctions. However, larger, prospective trials in younger patients are necessary to solve this question.

## Limitations

In the present study, we retrospectively observed the history of myocardial infarctions in a group of 1000 patients. Fatal cardiac events could not be considered in this study. The exact coronary status was not included in the analysis because coronary catheterisation was not performed in the OSA patients. In these patients the diagnosis of coronary heart disease was established by patient recall and searching of patients documents and thus it is not as reliable as the clinical diagnosis available in the CAR group. A further limitation of our work is the two-group ascertainment and the joint analysis. In addition the total number of patients is comparatively small for genetic association studies and therefore the power to detect an association may be low.

## Conclusion

In presence of other risk factors, the CYP2J2 G-50T failed to show a significant role in the development of myocardial infarction. However, since our result is close to the border of significance and because positive results were found in a younger study group, further research is necessary. Prospective studies with on average younger patients, defined methods and sufficient follow-up periods, observing nonfatal and fatal coronary events should be performed in the future to clarify this question.

## Competing interests

The authors declare that they have no competing interests.

## Authors' contributions

JB and DB carried out the molecular genetic studies and drafted the manuscript and are equally contributing to this work They were also involved in the study design and statistical analysis. MS and JTE supervised the molecular genetic studies, CH and HN were substantially involved in patient- and data collection. THL supervised the statistical analysis and AM participated the study design and coordination and helped to draft the manuscript.

## Pre-publication history

The pre-publication history for this paper can be accessed here:


